# Antimicrobial treatment in invasive infections caused by *Gordonia bronchialis*: systematic review

**DOI:** 10.3389/fmed.2024.1333663

**Published:** 2024-03-06

**Authors:** Radica Zivkovic Zaric, Petar Canovic, Milan Zaric, Marko Vuleta, Katarina Vuleta Nedic, Jovan Jovanovic, Nenad Zornic, Jelena Nesic, Marko Spasic, Stefan Jakovljevic, Milena Ilic, Dalibor Jovanovic, Zeljko Todorovic, Petar Arsenijevic, Miroslav Sovrlic, Jasmina Milovanovic

**Affiliations:** ^1^Department of Pharmacology and Toxicology, Faculty of Medical Sciences, University of Kragujevac, Kragujevac, Serbia; ^2^University Clinical Center Kragujevac, Kragujevac, Serbia; ^3^Department of Biochemistry, Faculty of Medical Sciences, University of Kragujevac, Kragujevac, Serbia; ^4^Department of Cardiology, General Hospital Dragisa Misovic, Belgrade, Serbia; ^5^Department of Internal Medicine, Faculty of Medical Sciences, University of Kragujevac, Kragujevac, Serbia; ^6^Department of Nuclear Medicine, Faculty of Medical Sciences, University of Kragujevac, Kragujevac, Serbia; ^7^Department of Surgery, Faculty of Medical Sciences, University of Kragujevac, Kragujevac, Serbia; ^8^Department of Pathology, Faculty of Medical Sciences, University of Kragujevac, Kragujevac, Serbia; ^9^Department of Gynecology and Obstetrics, Faculty of Medical Sciences, University of Kragujevac, Kragujevac, Serbia; ^10^Faculty of Medical Sciences, Center for Harm Reduction of Biological and Chemical Hazards, University of Kragujevac, Kragujevac, Serbia

**Keywords:** *Gordonia bronchialis*, antimicrobial treatment, invasive infections, vancomycin, ciprofloxacin

## Abstract

**Background:**

Corynebacterium, Nocardia, Rhodococcus, Mycobacterium, as well as Gordonia genera belongs to the genus Gordonia, Actinomycetia class. *Gordonia bronchialis* is a nitrate-reducing, urease-producing, non-motile, force aerobe with a rod-like figure that is known to arrangement into sessile, cord-like groups. This systematic review aimed to establish whether and what invasive infections in humans were caused by *Gordonia bronchialis*, and to evaluate outcomes of administered antibiotic treatment.

**Methods:**

We have registered this systematic review in PROSPERO database of systematic reviews and meta-analyses with the number CRD42022369974.

**Results:**

A total of 24 publications were included (22 case reports and two case series) with 28 individual cases. The oldest patients had 92 years, and the youngest patients had 13 years. Clinical signs of infection were present in six patients (21%). All isolates were susceptible to ciprofloxacin, imipenem, and amikacin. Vancomycin was the most frequently used antibiotic with nine cases followed by ciprofloxacin, ceftriaxone, and amoxicillin/clavulanic acid.

**Conclusion:**

Although there are no standardized recommendations to date, successful treatment with a favorable outcome has most often been carried out with fluoroquinolones, vancomycin with or without aminoglycosides, as well as carbapenems.

## Introduction

Corynebacterium, Nocardia, Rhodococcus, Mycobacterium, as well as Gordonia genera belongs to the genus Gordonia, Actinomycetia class (1). Tsukamura in 1971 give a name for this genus, initially called Gordona, because he wanted to pay tribute to the American bacteriologist Ruth E. Gordon (2). Infections with Gordonia spp. (previously categorized as Rhodococcus spp.) have been linked with medical involvements, and main pathogens include *Gordonia bronchialis*, *Gordonia sputi*, and *Gordonia terrae* (1). Fellows of the genus Gordonia are aerobic, Gram-positive, catalase-positive, non-motile, nocardioform actinomycetes that are weakly acid-fast. *Gordonia bronchialis* also is a nitrate-reducing, urease-producing, non-motile, force aerobe with a rod-like figure that is known to arrangement into sessile, cord-like groups. It owns lipoglycan similar to Mycobacterium, which serves as a key factor of virulence (2, 3).

Some Gordonia species (including *Gordonia bronchialis*) isolated from clinical samples are known to be opportunistic human pathogens initiating secondary infections in immunosuppressive and immunocompetent humans (4). *Gordonia bronchialis* (formerly known as Rodococcus bronchialis) has been isolated from environmental samples and prosthetic ingredients and has been reported as an opportunistic infection in humans. Microbiologically it is difficult to recognize *Gordonia bronchialis*. Speciation of *G. bronchialis* is achieved via 16S rRNA sequencing and matrix-assisted laser desorption/ionization time-of-flight (MALDI-TOF) mass spectrometry. Other systems are currently ineffective due to database limitations (5–7). This systematic review aimed to establish whether and what invasive infections in humans were caused by *Gordonia bronchialis*, to evaluate outcomes of administered antibiotic treatment as well as to observe the current situation regarding the sensitivity of the bacteria *G. bronchialis* to antibiotics.

## Methods

We have registered this systematic review at PROSPERO database of systematic reviews and meta-analyses with number CRD42022369974 (8).

Studies to be included in this systematic review firstly must to fulfill the following inclusion criteria: (1) type of study—clinical trial, observational study (cohort and cross-sectional), case series, and case report; (2) characteristics of individuals—patients of any age and gender harboring *Gordonia bronchialis* as the only bacteria in body fluids or tissues, from where it was isolated and identified by MALDI-TOF mass spectrometry and/or by 16 s RNA sequencing. The exclusion criteria were: (1) review articles just citing infection caused by *G. bronchialis*, (2) isolation of *G. bronchialis* from skin or mucous surfaces without evidences of infiltration through epithelial lining, (3) cases of *G. bronchialis* infections in nonhuman species, and (4) studies with partial data.

Studies were searched from electronic databases and collection of journal articles and books at University Library, University of Kragujevac, Kragujevac, Serbia. Electronic searches of available studies were conducted in MEDLINE (PubMed coverage from 1966 to present), EBSCO (Discovery Service, coverage from 1944 to present) The Cochrane Central Register of Controlled Trials (Central) (Wiley Online Library, coverage from 1966 to present), and SCIndeks, Scopus, Google Scholar and in Registry of clinical studies with human participants, ClinicalTrials.gov. Electronic records were searched self-sufficiently for relevant studies by four authors: RZ, MZ, PC, and JM. The search strategy made by the researcher RZ for the MEDLINE database was the most extensive: (Gordonia [All Fields] AND bronchialis [All Fields] AND ((“infection”[MeSH Terms] OR “infection”[All Fields]) OR (“infection”[MeSH Terms] OR “infection”[All Fields] OR “infections”[All Fields]) OR “bloodstream infection”[All Fields] OR (“endocarditis”[MeSH Terms] OR “endocarditis” [All Fields]) OR (“sepsis”[MeSH Terms] OR “sepsis”[All Fields]) OR pathogen [All Fields] OR (“bacteraemia” [All Fields] OR “bacteremia”[MeSH Terms] OR “bacteremia”[All Fields]) OR (“bacteraemia”[All Fields] OR “bacteremia”[MeSH Terms] OR “bacteremia”[All Fields]) OR (“peritonitis”[MeSH Terms] OR “peritonitis” [All Fields]) OR (“osteomyelitis”[MeSH Terms] OR “osteomyelitis” [All Fields]) OR (“arthritis”[MeSH Terms] OR “arthritis”[All Fields]) OR (“patients”[MeSH Terms] OR “patients”[All Fields] OR “patient”[All Fields]) OR (“pneumonia”[MeSH Terms] OR “pneumonia”[All Fields]) OR (“bronchitis”[MeSH Terms] OR “bronchitis”[All Fields]) OR (“sinusitis” [MeSH Terms] OR “sinusitis”[All Fields]) OR (“abscess”[MeSH Terms] OR “abscess”[All Fields])). Saved articles were first evaluated from the title and abstract for eligibility, and if it was not conceivable, the full text of manuscripts was examined. If all authors agreed that retrieved manuscript fulfills eligibility criteria, that manuscript would be involved in further review process. If the eligibility of study has not been approved by all authors, senior author (RZ) makes the final choice.

The data were taken out by three investigators independently (RZ, MZ, and PC) and collected in the final extraction table by another investigator (JM).

Risk of bias was evaluated by two investigators individualistically (MZ and PC), and the senior investigator (RZZ) made finishing assessment. The following bases of bias were evaluated: (1) reporting bias and (2) attrition bias. Reporting bias is related to the adequacy of the displayed data in terms of detail, and attrition bias is important for whether we finally tracked down what happened to the patient (9).

From all dates, the following outcomes were categorical: sex of patients, method for *G. bronchialis* identification (biochemical methods or MALDI TOF mass spectrometry or 16 s RNA sequencing), variations in laboratory parameters of an tissue role suggestive of that tissue infection, consequences of antibiotic treatment (cure rate and mortality), adverse events rate and type, antibiotics used, and resistance frequency of *G. bronchialis* to antibiotics and morphological diagnostics which established aggressive infection (NMR, ultrasound, etc.). Following outcomes were continuous: study duration, age of patients, number of patients, maximal serum level of C-reactive protein during the disease and maximal white blood cell count during the disease.

## Results

**Figure 1:**
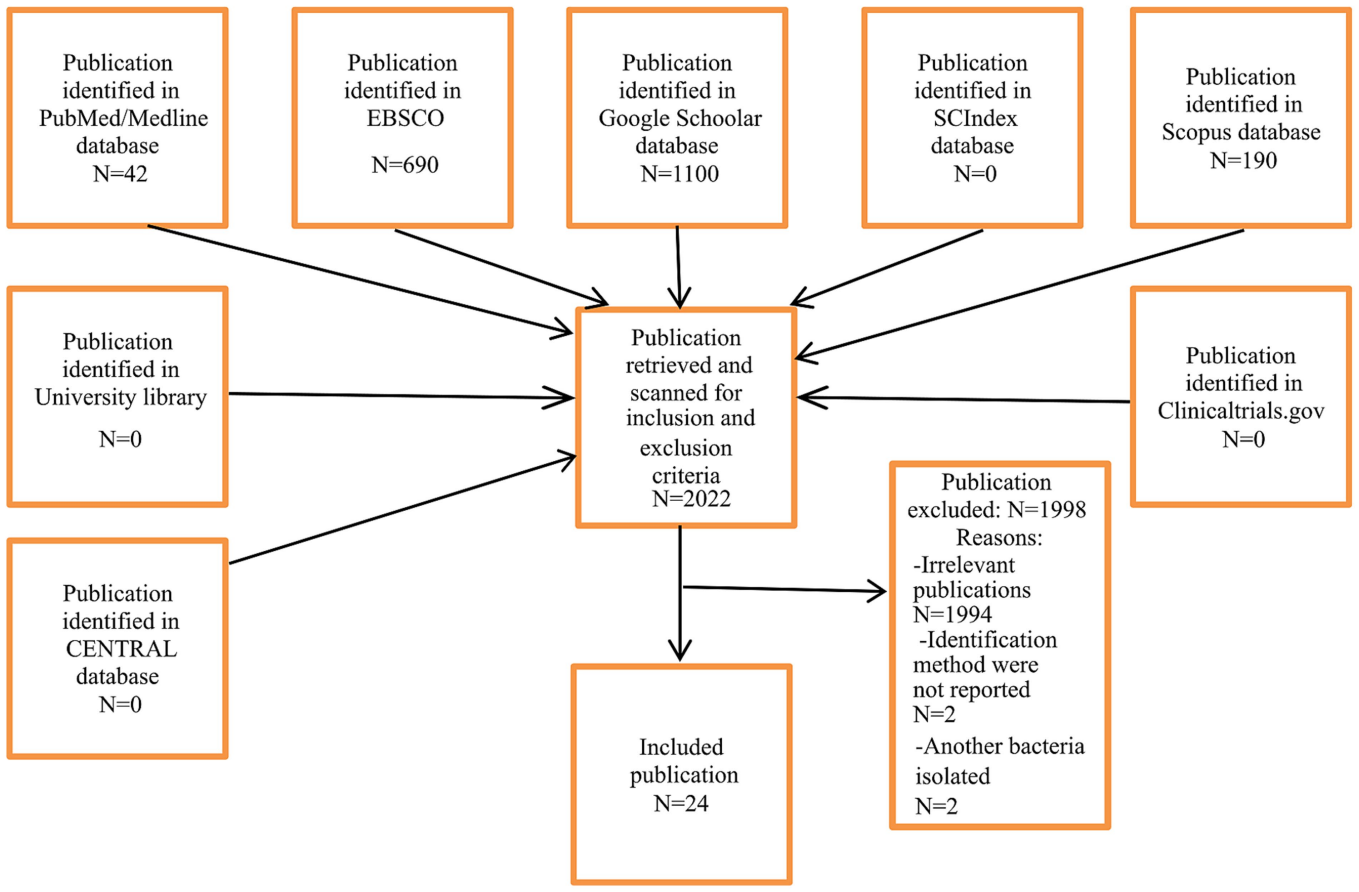
Selection of studies.

In Figure 1, search results are represented. A total of 24 publications were included (22 case reports and two case series) with 28 individual cases. The oldest patients had 92 years, and the youngest patients had 13 years. Fourteen individuals were females, vs. 12 male individuals. In two cases, gender was not reported.

Most of the cases were represented in the United States of America (*n* = 10; 35%) followed by Japan (*n* = 6; 21%) and Spain (*n* = 4; 14%). Other cases were noted in France, Poland, Lithuania, Korea, Canada, Qatar, Singapur, and New Zeland. All patients but one was hospitalized. The longest duration of the study was 10 years. Characteristics of individual cases were shown in Table 1.

**Table 1:** Characteristics of cases included in the study.

Publication ID	Study design	Attrition/reporting bias	Age	Gender	Site of isolation *G. bronchialis*	Morphological diagnosis of infection	AB used for therapy	Outcomes of AB treatment
Akrami et al. (10)	Case report	Low/Low	68	M	Wound tissue	NR	Vancomycine; Ceftaroline	Cured
Alnajjar et al. (11)	Case report	Low/Low	46	M	Blood	Bacteremia	Ceftriaxone	Cured
Alvarez et al. (12)	Case report	High/Low	50	F	Pus from abscess	Abscess	PO amoxicillin-clavulanate	NR
Ambesh et al. (13)	Case report	High/High	74	M	Wound tissue	Osteomyelitis	IV vancomycine and meropenem	NR
Aoyama et al. (14)	Case series	High/High	70	M	Peritoneal fluid	Peritonitis	NR	NR
Aoyama et al. (14)	Case series	High/High	51	F	Sputum	Pneumonia	NR	NR
Aoyama et al. (14)	Case series	High/High	NR	NR	Sputum	Pneumonia	NR	NR
Aoyama et al. (14)	Case series	High/High	NR	NR	Sputum	Pneumonia	NR	NR
Aoyama et al. (14)	Case series	High/High	77	M	Sputum	Pneumonia	NR	NR
Bayo et al. (15)	Case report	Low/Low	88	F	Blood	Endocarditis	IV ceftriaxone and ciprofloxacin	Cured
					Pacemaker			
Bruno et al. (15)	Case report	Low/Low	13	F	Peritoneal fluid	Peritonitis	IP vancomycin; PO ciprofloxacine	Cured
Brust et al. (16)	Case series	High/High	67	M	Blood	Bacteriemia	NR	NR
Chang et al. (17)	Case report	Low/Low	69	F	Wound tissue	Osteomyelitis	IV vancomycin and cefotetan; imipenem	Cured
Choi et al. (18)	Case report	Low/Low	63	F	Vitreous sample	Endophtalmitis	IT injection of amikacin;IT ceftazidime was administered to the left eye, and PO moxifloxacin	Partially recovery
Davidson et al. (6)	Case report	Low/Low	26	F	Wound tissue	NR	Amoxicilin clavunate	Cured
Franczuk et al. (19)	Case report	Low/Low	70	M	Sputum	Pneumonia	Cotrimoxazole	Cured
James et al. (20)	Case report	Low/Low	27	M	Sputum	Pneumonia	Levofloxacin	Cured
Jonson et al. (21)	Case report	Low/Low	52	F	Pleural fluid and blood	Pleuritis	IV vancomycin and ceftazidime; IV cotrimoxazole and imipenem-cilastatin; PO cotrimoxazole; and PO ciprofloxacin and minocycline	Cured
						Bacteriemia		
Mc Kormic et al. (1)	Case report	Low/Low	56	F	Blood	Bacteriemia	Cotrimoxazole and imipenem	Cured
Nakahama et al. (22)	Case report	Low/Low	68	M	Sputum	Pneumonia	None	Cured
Nwaedozie et al. (23)	Case report	Low/Low	81	M	Tissue and bone sample	Osteomyelitis	IV vancomycin and cefazolin; PO amoxicillin/clav ulanate; IV ceftriaxone; and IV ampicillin sulbactam	Cured
Rodriguez Lozano et al. (24)	Case report	Low/Low	64	F	Wound tissue	Sternal wound infection	IV clindamycin and ceftazidime; IV imipenem and ciprofloxacin; IV teicoplanin plus ciprofloxacin; and rifampin	Cured
Siduiqqi et al. (25)	Case report	Low/Low	22	F	Bone sample	Osteomyelitis	PO cotrimoxazole; PO amoxicilin clavunat; and IV vancomycin	Cured
Sng et al. (26)	Case report	Low/Low	58	F	Blood	Bacteremia	IV vancomycin and ceftriaxone followed by PO amoxicillin-clavulanate	Cured
Sukackiene et al. (27)	Case report	Low/Low	32	M	Peritoneal fluid	Peritonitis	IP vancomycin and gentamycin; PO ciprofloxacine	Cured
Tite’cat et al. (28)	Case report	Low/Low	92	M	Pacemaker	Endocarditis	IV piperacillin-tazobactam and daptomycin; PO amoxicillin	Cured
Vasquez et al. (29)	Case report	Low/Low	76	F	Tissue and bone sample	Osteomyelitis	IV ceftriaxone; PO ciprofloxacin	Cured
Werno et al. (30)	Case report	Low/High	43	F	Pus from abscess	Abscess	IV penicillin and flucloxacillin; PO amoxicillin-clavulanate and metronidazole; and PO doxycycline and clindamycin	Cured

F-Female.

M-Male.

NR- Not reported.

IV- Intravenously.

PO-Per os.

*Gordonia bronchialis* was most frequently isolated from sputum (*n* = 7; 25%) as well as from wound tissue (*n* = 7; 25%), followed by blood (*n* = 6; 21%), peritoneal fluid (*n* = 3; 10%), bone sample (*n* = 3; 10%), pus (*n* = 3; 10%), and pacemaker (*n* = 2; 7%). In one case, each *G. bronchialis* was isolated from pleural fluid, corpus vitreum. Only gene sequencing was the most common method for *G. bronchialis* identification (*n* = 13, 46%). A combination of MALDI-TOF mass spectrometry with gene sequencing was used in 13 cases (46%) as well as a combination of biochemical methods with gene sequencing in two cases (7%).

Clinical signs of infection were present in six patients (21%) with a patients with maximal level of body temperature at 40°C. The maximum level of white blood cell count and C reactive protein was noted in six patients (21%). Results of morphological diagnostics (e.g., CT, ultrasound) were represented in 16 patients (57%) with the following findings: endocarditis, pneumonia, and osteomyelitis.

**Figure 2:**
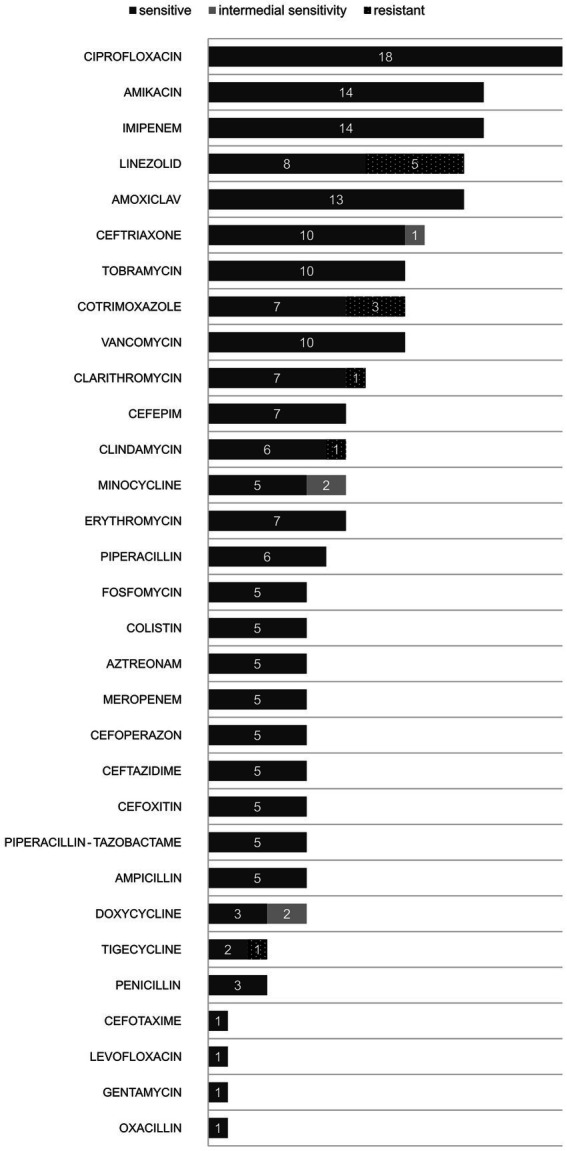
Susceptibility to antibiotics of *Gordonia bronchialis*.

Sensitivity to antibiotics was tested in 21 patients (75%). The results are shown in Figure 2. The most frequently tested antibiotics were ciprofloxacin, followed by imipenem and amikacin. All isolates were susceptible to ciprofloxacin (*n* = 18; 64%), imipenem (*n* = 14; 50%) as well as amikacin (*n* = 14; 50%). *Gordonia bronchialis* showed some resistance to linezolid (5 vs. 8 susceptible isolates), clarithromycin (1 vs. 7 susceptible isolates), as well as tygeciklin (1 vs. 3 susceptible isolates) and cotrimoxazole (3 vs. 7 susceptible isolates).

Antibiotic therapy for individual cases is shown in Table 1. The majority of the patients had a combination of antibiotics (*n* = 16, 57%). In five cases, only one antibiotic was used. In six cases, antibiotic treatment was not reported and in one case antibiotic treatment was not needed. Vancomycin was the most frequently used antibiotic with nine cases (32%) followed by ciprofloxacin (*n* = 8; 28%), ceftriaxone (*n* = 5; 17%), and amoxicillin/clavulanic acid (*n* = 5; 17%). Cotrimoxazole (*n* = 4; 24%), imipenem (*n* = 3; 10%), and ceftazidime (*n* = 3; 10%) also were used. All patients but one recovered completely. There were no cases with fatal outcomes. In one case, cotrimoxazole caused severe dyspnea and rash.

## Discussion

The results of our study showed that *G. bronchialis* causes numerous, diverse, and serious infections encompassing pneumonia and osteomyelitis as the most frequent, followed by bacteremia, endocarditis, and peritonitis, etc. Human samples for the isolation of *G. bronchialis* were collected from sputum, wound tissue, blood, bone, peritoneal and pleural fluid, corpus vitreum, and medical device (pacemaker), while its identification was confirmed using gene sequencing method and/or MALDI-TOF-MS analysis. Sensitivity to antibiotics, such as ciprofloxacin, imipenem, and amikacin was proven in the largest number of cases. Treatment of infection caused by *G. bronchialis* was primarily based on combined antibiotic therapy, while one anti-infective drug was administered in two patients. The most frequently used antibiotics were vancomycin and ciprofloxacin. All patients underwent hospital treatment and recovered completely after therapy (one without treatment), except for one patient who was partially cured. There were no mortality-related outcomes. Even though this bacterium does not currently lead to death, the importance of our review is that the resistance of bacteria to antibiotics is constantly increasing. This creates the problem of life-threatening infections because it has been shown that more people die from infections with multi-resistant bacteria than from homicide, HIV infections, etc. The problem is that the treatment costs are much higher. We need to get on the road to that by treating the infection adequately right away so that problems do not arise over time (31–33). The significance of our manuscript is that clinicians will be sure how to treat infection adequately caused by *G. bronchialis*, and there is less chance that resistance will develop at all in the future.

Overall, infections due to *G. bronchialis* were most often represented among patients associated with cardiac surgical intervention (usually coronary artery bypass grafting) and/or the implantation of prosthetic materials (cardiac pacemaker, indwelling catheters, prosthetic valve, intraocular lens implant, and bioresorbable polymer screw). Out of a total of 28 patients included in our systematic review, only two patients did not undergo surgery or insertion of prosthetic material. No data were available for five patients.

Treatment of infections caused by *G. bronchialis* was mandatory based on pharmacotherapy in all patients, except one.

A review of the literature related to *G. bronchialis* infections in humans, clearly shows the difficulties associated with its identification, even today. *Gordonia bronchialis* was often misidentified as Nocardia, Rhodococcus, or non-tuberculosis mycobacteria because of their morphological similarity and production of mycolic acids, or dismissed as commensal when conventional biochemical or microbiological methods were used (34). Although there is clear evidence that genetic 16S rRNA sequencing and MALDI-TOF-MS methods ensure accurate identification of *G. bronchialis* to species level, microbiological laboratories are usually not adequately equipped and standard laboratory tests are often insufficient (34–36). As additional phylogenetic markers for the identification of Gordonia strains, the GyrB, secA, and hsp65 genes are the most commonly used today (37). A lack of significant attention to the pathogenic role of *G. bronchialis*, as primarily environmental bacteria, in causing both local and serious systemic infections probably contributes to this Mormeneo et al. (2). A longer incubation period of 3–4 days to the isolation of *G. bronchialis* strains also may give false negative results or it may be missed in clinical samples (16, 38). Furthermore, the ability of this bacteria to create a biofilm, due to the production of Gordon, an acidic polysaccharide with cell aggregation-inductive activity, that has adhesive properties to the hydrophobic surfaces, such as the previously mentioned prosthetic materials, results in prolonged use of antibiotics in patients and consequently the increase of bacterial resistance (2, 16, 37, 39).

Although pneumonia caused by *G. bronchialis* is the most common diagnosis in our systematic review, only three patients have a complete medical history (16, 17, 19). Antibiotic drugs used in the treatment of pneumonia were levofloxacin and sulfamethoxazole/trimethoprim, as monotherapy. Levofloxacin is known to have a broad spectrum of activity against many causative agents of community-acquired pneumonia. Its favorable pharmacokinetic properties, such as good absorption, bioavailability, and maintenance of drug concentration at the site of infection with good tissue penetration enable its effectiveness when used as oral monotherapy (40).

Cotrimoxazole has been used alone or in combination with imipenem, oral, and intravenous, to treat various infections with *G. bronchialis*, such as pneumonia, tibial osteomyelitis, and bacteremia. In contrast, some literature data suggest the poor activity of sulfamethoxazole/trimethoprim against Gordonia species (≤65% of isolates) (5, 41).

Peritonitis associated with *G. bronchialis* infection has been reported in two patients who underwent chronic peritoneal dialysis, an adult and a 13-year old girl. Empirical antibiotic treatment included intra-peritoneal doses of vancomycin in combination with ceftazidime or gentamicin.

Fluoroquinolones, aminoglycosides, third generation cephalosporins, and amoxicillin/clavulanate are effective in treating *G. bronchialis* infections, but bacterial eradication from PD catheters was not achieved, probably due to an adhesive biofilm that reduces drug penetration (12, 26). These antimicrobials have shown well *in vitro* activity against Gordonia species (>90% of tested isolates), including vancomycin, in other studies as well (12, 42). Ciprofloxacin showed excellent activity against *G. bronchialis* (all tested isolates in this systematic review), good oral bioavailability, and together with vancomycin, it is the most commonly used antibiotic in the treatment of infections caused by *G. bronchialis*. Moreover, the drug achieves an optimal concentration in bones and soft tissues even after oral administration, which is an effective treatment in the treatment of osteomyelitis and soft tissue infections (22, 43). It has been used rarely as monotherapy, most often in combination with another anti-infective drug such as amoxicillin/clavulanate, ceftriaxone, imipenem, teicoplanin, minocycline, rifampin, and only in one case as triple antibiotic therapy with teicoplanin and rifampin, for treating various infections related to *G. bronchialis* as the main pathogen (2, 12, 18, 21, 25).

Vancomycin has been used in many cases as the first empiric therapy, alone or with other antibiotics, but also as the final, target therapy after identification of *G. bronchialis*. Vancomycin was administered in co-therapy with cephalosporins (ceftazidime, cefazolin, or cefotetan), gentamicin, or meropenem in the treatment of various *G. bronchialis* infections, as peritonitis, osteomyelitis or bacteremia following pleuritis. However, literature data suggested that almost 11% of Gordonia species showed resistance to vancomycin (5).

Bacteremia due to *G. bronchialis* infection is rarely represented, and the most common causative agents were *G. sputi* and *G. terrae* (18, 44). The carbapenem group of antibiotics, primarily imipenem, was frequently used in the treatment of bacteremia and osteomyelitis (a total of five cases) caused by *G. bronchialis*. Meropenem was effective in combination with vancomycin in one case, while imipenem was used alone or in combination with sulfamethoxazole/trimethoprim or ciprofloxacin with notable favorable outcomes in treated patients. All tested isolates in our review were susceptible to imipenem (almost 60% of the total number) which makes it a suitable drug for the treatment of these infections. Similarly, almost all tested isolates of Gordonia species were susceptible to imipenem in the review by Aoyama et al. (14).

The optimal antibiotic treatment in these cases was between 6 and 12 weeks to avoid the possibility of relapse in the patient (20). This is supported by findings that prolonged antibiotic use is necessary for the effective treatment of osteomyelitis (45).

Although there are no standardized recommendations to date, successful treatment with a favorable outcome has most often been carried out with fluoroquinolones, vancomycin with or without aminoglycosides, carbapenems, third-generation cephalosporins. However, it should be mentioned that *G. bronchialis* showed some resistance to linezolid, clarithromycin, tigecycline, and cotrimoxazole. One case of allergic reaction to co-trimoxazole has been recorded.

The main limitation of our systematic literature review is the small number of studies. We believe this is related to the still underutilized precise laboratory methods for the correct identification of *G. bronchialis* at the species level.

*Gordonia bronchialis* infections should be taken seriously, because if not, it can lead to a high degree of resistance to antibiotics. The treatment of *G. bronchialis* infections should include fluoroquinolones, vancomycin with or without aminoglycosides, and carbapenems. Cotrimoxazole as well as linezolid should be avoided, because in some cases *G. bronchialis* shows resistance to those antibiotics.

## Data availability statement

The original contributions presented in the study are included in the article/supplementary material, further inquiries can be directed to the corresponding author.

## Author contributions

RZZ: Conceptualization, Methodology, Visualization, Writing – original draft. PC: Investigation, Resources, Writing – original draft. MZ: Conceptualization, Methodology, Writing – review & editing. MV: Formal Analysis, Validation, Writing – original draft. KVN: Formal analysis, Investigation, Writing – review & editing. JJ: Validation, Writing – original draft. NZ: Conceptualization, Supervision, Writing – original draft. JN: Data curation, Supervision, Writing – original draft. MS: Supervision, Validation, Writing – review & editing. SJ: Methodology, Validation, Writing – original draft. MI: Methodology, Investigation, Writing – review & editing. DJ: Data curation, Writing – review & editing. PA: Resources, Supervision, Writing – original draft. MSo: Conceptualization, Validation, Writing – review and editing. JM: Software, Writing – original draft preparation, Writing – review and editing.
